# Bis(2-amino­benzimidazolium) sulfate monohydrate

**DOI:** 10.1107/S2414314622001729

**Published:** 2022-02-22

**Authors:** Adrian Peña Hueso, Adriana Esparza Ruiz, Angelina Flores Parra

**Affiliations:** a Departamento de Química, Centro de Investigación y de Estudios Avanzados del Instituto Politécnico Nacional, 14-740, Ciudad de Mexico, CP 07000, Mexico; b Facultad de Ingeniería Química, Universidad Autónoma de Yucatán, Periférico Norte Km 33.5, Tablaje Catastral 13615, Chuburna de Hidalgo Inn, Mérida, Yucatan, C.P 97203, Mexico; University of Aberdeen, Scotland

**Keywords:** crystal structure, 2-amino­benzimidazolium, sulfate

## Abstract

In the title hydrated mol­ecular salt, 2C_7_H_8_N_3_^+^·SO_4_^2−^·H_2_O, the components are linked by numerous N—H⋯O and O—H⋯O hydrogen bonds.

## Abstract 2

The components of the title mol­ecular salt are linked by N—H⋯O and O—H⋯O hydrogen bonds.

## Graphical abstract

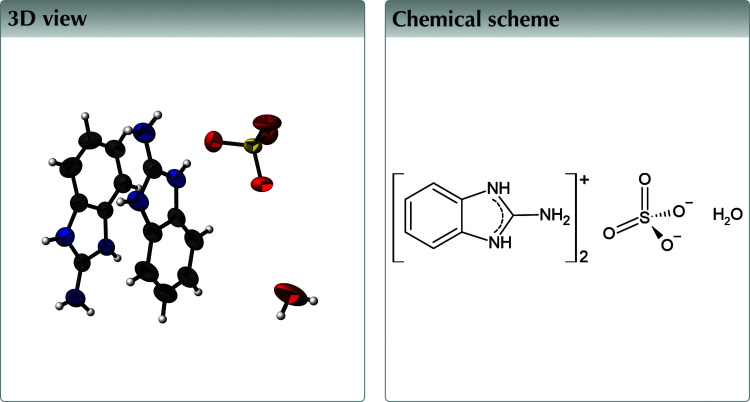

## Structure description

2-Amino­benzimidazole has been used for the synthesis of a series of sulfur heterocycles such as 9*H*-3-thia-1,4a,9-tri­aza-fluorene-2,4-di­thione (**1**): its potassium thiol­ate salt was used to prepare metal coordination compounds (Peña-Hueso *et al.*, 2008 ▸), and is the precursor of the title compound. When compound **1** is dissolved in dimethyl sulfoxide and strong acids are added, instead of producing the protonated derivative, the thia­diazine ring breaks down, producing 2-amino­benzimidazolium sulfate (**2**): its crystal structural features are the subject of the present paper.

**Figure 1:**
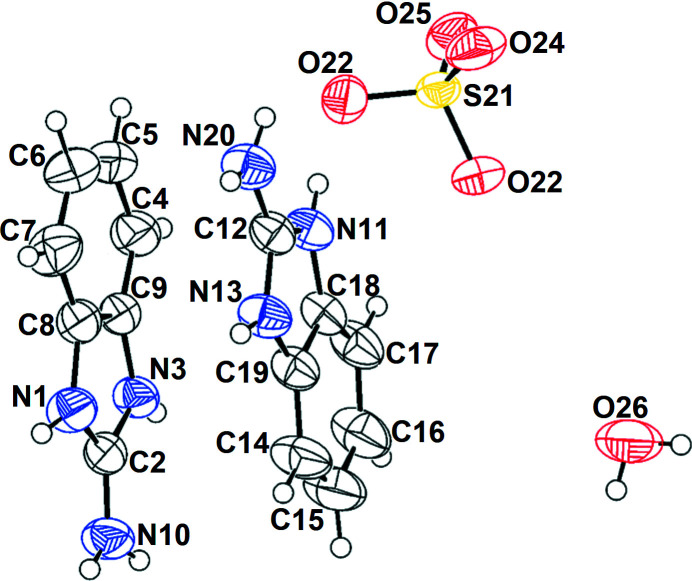
The mol­ecular structure of **2** showing displacement ellipsoids drawn at the 50% probability level

Compound **2** is formed by the transfer of two protons from sulfuric acid to the heterocycle: the crystal has two 2-amino­benzimidazolium cations, one sulfate anion and one water mol­ecule in its asymmetric unit (Fig. 1 ▸). There is a small asymmetry in the S—O bond lengths of the SO_4_^2–^ ion from 1.4596 (16) to 1.4723 (15) Å, probably caused by the hydrogen bonds around the anion (Gagné & Hawthorne, 2018 ▸). Two benzimidazolium cations are stacked in a head-to-tail way, with a distance between C9 of one mol­ecule and C18 of another of 3.441 (3) Å.

**Figure 2:**
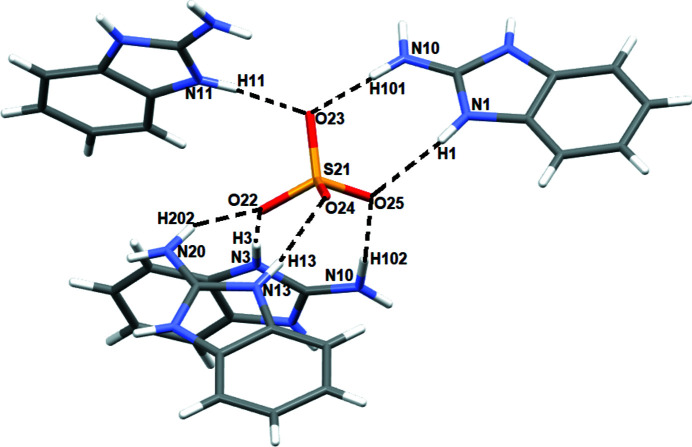
Hydrogen-bond environment around the sulfate anion.

**Figure 3:**
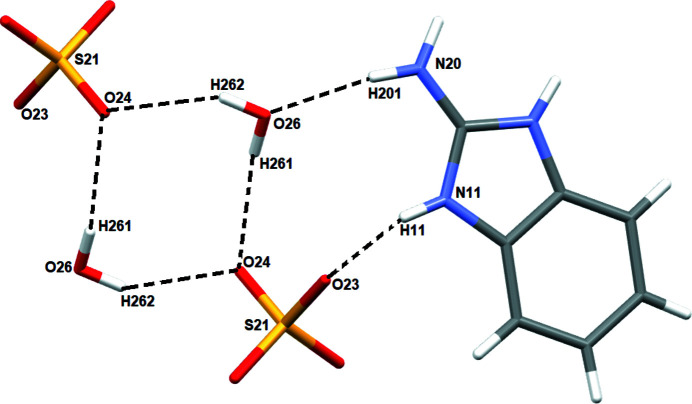
Network of hydrogen bonds (dashed lines) involving the water mol­ecules and sulfate ions.

**Figure 4:**
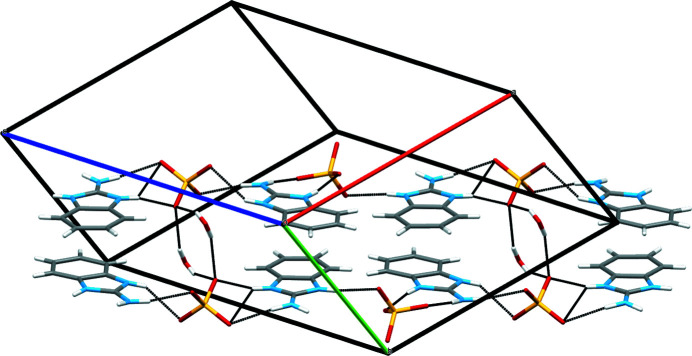
The unit-cell packing showing [010] ribbons of cations linked by sulfate anions.

The sulfate ion accepts seven N—H⋯O hydrogen bonds from four adjacent benzimidazolium cations and one O—H⋯O link from a water mol­ecule (Table 1 ▸, Fig. 2 ▸). The water mol­ecule accepts one N—H⋯O hydrogen bond and forms two O—H⋯O links to two SO_4_^2–^ ions (Fig. 3 ▸). In the extended structure, the benzimidazolium cations form parallel ribbons propagating in the [010] direction (Fig. 4 ▸).

The first crystal structure of a 2-amino­benzimidazolium salt was reported with the nitrate anion (Bats *et al.*, 1999 ▸) and a related structure with hydrogen sulfate as the counter-ion is also known (You *et al.*, 2009 ▸).

## Synthesis and crystallization

The decomposition of 9*H*-3-thia-1,4a,9-tri­aza-fluorene-2,4-di­thione with dilute aqueous H_2_SO_4_ in DMSO afforded the title compound **2**, m.p. 287–289°C. IR (KBr), ν (cm^−1^): 3285 (N—H), 1682 (C=N), 1520 (C=C), 1478 (C—N). NMR (DMSO-*d*_6_, p.p.m.) δ ^1^H: 7.27 (H4, H7); 7.09 (H5, H6); 13.18 (N1—H, N3—H); 8.70 (NH_2_). δ ^13^C: 152.1 (C2); 111.8 (C4, C7); 123.4 (C5, C6); 130.4 (C8, C9). δ ^15^N: −257.1 (N1, N3); −312.9 (N10). Analysis calculated (%) for C_16_H_16_N_6_SO_5_: C, 43.97; H, 4.74; N, 21.98. Found: C, 43.50; H, 4.80; N, 21.80. The chemical shifts of C2 (152.1 p.p.m.), C8 and C9 (130.4 p.p.m.) in the ^13^C NMR spectrum indicate that the endocyclic nitro­gen atoms are protonated, in agreement with the crystal structure.

## Refinement

Crystal data, data collection and structure refinement details are summarized in Table 2 ▸.

## Supplementary Material

Crystal structure: contains datablock(s) I. DOI: 10.1107/S2414314622001729/hb4397sup1.cif

Structure factors: contains datablock(s) I. DOI: 10.1107/S2414314622001729/hb4397Isup2.hkl

Supporting information file. DOI: 10.1107/S2414314622001729/hb4397Isup3.cml

CCDC reference: 1810894

Additional supporting information:  crystallographic information; 3D view; checkCIF report

## Figures and Tables

**Table 1:** Hydrogen-bond geometry (Å, °)

*D*—H⋯*A*	*D*—H	H⋯*A*	*D*⋯*A*	*D*—H⋯*A*
N1—H1⋯O25^i^	0.81 (4)	2.25 (3)	2.946 (3)	144 (3)
N3—H3⋯O22^ii^	0.83 (3)	1.93 (3)	2.749 (3)	172 (3)
N11—H11⋯O23	0.85 (4)	1.96 (4)	2.786 (4)	166 (3)
N13—H13⋯O24^iii^	0.83 (4)	1.91 (4)	2.720 (3)	165 (3)
N10—H101⋯O23^i^	0.87 (4)	2.03 (4)	2.894 (5)	169 (3)
N10—H102⋯O25^ii^	0.89 (3)	2.00 (3)	2.890 (3)	175 (3)
N20—H201⋯O26^iii^	0.84 (3)	2.04 (3)	2.853 (4)	165 (3)
N20—H202⋯O22^iii^	0.93 (4)	2.09 (4)	2.973 (4)	157 (3)
O26—H261⋯O24^iv^	0.80 (7)	2.22 (7)	2.983 (4)	160 (7)
O26—H262⋯O24^v^	0.80 (7)	2.14 (7)	2.860 (4)	150 (6)
C17—H17⋯O22	0.95	2.56	3.272 (3)	132

Symmetry codes: (i) 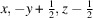 ; (ii) 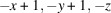 ; (iii) 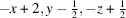 ; (iv) 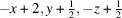 ; (v) 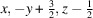 .

**Table 2:** Experimental details

Crystal data	
Chemical formula	2C_7_H_8_N_3_^+^·SO_4_^2−^·H_2_O
*M*_r_	382.4
Crystal system, space group	Monoclinic, *P*2_1_/*c*
Temperature (K)	293
*a*, *b*, *c* (Å)	12.1115 (2), 10.6282 (2), 17.4772 (3)
β (°)	127.723 (1)
*V* (Å^3^)	1779.48 (6)
*Z*	4
Radiation type	Mo *K*α
μ (mm^−1^)	0.22
Crystal size (mm)	0.25 × 0.25 × 0.17

Data collection	
Diffractometer	Nonius KappaCCD
No. of measured, independent and observed [*I* > 3.0σ(*I*)] reflections	9132, 4563, 2429
*R*_int_	0.04
(sin θ/λ)_max_ (Å^−1^)	0.675

Refinement	
*R*[*F* ^2^ > 2σ(*F* ^2^)], *wR*(*F* ^2^), *S*	0.041, 0.050, 1.03
No. of reflections	2429
No. of parameters	265
H-atom treatment	H atoms treated by a mixture of independent and constrained refinement
Δρ_max_, Δρ_min_ (e Å^−3^)	0.25, −0.31

Computer programs: *COLLECT* (Nonius, 2001 ▸), *DENZO*/*SCALEPACK* (Otwinowski & Minor, 1997 ▸), *SHELXS97* (Sheldrick, 2008 ▸), *CRYSTALS* (Betteridge *et al.*, 2003 ▸) and *CAMERON* (Watkin *et al.*, 1996 ▸).

## full crystallographic data

### Crystal data

**Table:**

2C_7_H_8_N_3_^+^·SO_4_^2^^−^·H_2_O	*F*(000) = 800
*M**_r_* = 382.4	*D*_x_ = 1.427 Mg m^−^^3^
Monoclinic, *P*2_1_/*c*	Mo *K*α radiation, λ = 0.71073 Å
Hall symbol: -P 2ybc	Cell parameters from 4784 reflections
*a* = 12.1115 (2) Å	θ = 1–29°
*b* = 10.6282 (2) Å	µ = 0.22 mm^−^^1^
*c* = 17.4772 (3) Å	*T* = 293 K
β = 127.723 (1)°	Prism, colourless
*V* = 1779.48 (6) Å^3^	0.25 × 0.25 × 0.17 mm
*Z* = 4	

### Data collection

**Table:**

Nonius KappaCCD diffractometer	2429 reflections with *I* > 3.0σ(*I*)
Radiation source: Enraf Nonius FR590	*R*_int_ = 0.04
Graphite monochromator	θ_max_ = 28.7°, θ_min_ = 2.1°
Detector resolution: 9 pixels mm^-1^	*h* = −15→16
φ & ω scans	*k* = −14→14
9132 measured reflections	*l* = −23→23
4563 independent reflections	

### Refinement

**Table:**

Refinement on *F*	Primary atom site location: structure-invariant direct methods
Least-squares matrix: full	Secondary atom site location: difference Fourier map
*R*[*F*^2^ > 2σ(*F*^2^)] = 0.041	Hydrogen site location: difference Fourier map
*wR*(*F*^2^) = 0.050	H atoms treated by a mixture of independent and constrained refinement
*S* = 1.03	Method, part 1, Chebychev polynomial, (Watkin, 1994, *P*rince, 1982) [*w*eight] = 1.0/[A_0_*T_0_(x) + A_1_*T_1_(x) ··· + A_n-1_]*T_n-1_(x)] where A_i_ are the Chebychev coefficients listed belo*w* and x = *F* /*F*max Method = Robust Weighting (*P*rince, 1982) W = [*w*eight] * [1-(delta*F*/6*sigma*F*)^2^]^2^ A_i_ are: 0.914 0.838 0.564 0.170 0.849E-01
2429 reflections	(Δ/σ)_max_ = 0.0002
265 parameters	Δρ_max_ = 0.25 e Å^−^^3^
0 restraints	Δρ_min_ = −0.31 e Å^−^^3^

### Special details

**Table:**

Geometry. All esds (except the esd in the dihedral angle between two l.s. planes) are estimated using the full covariance matrix. The cell esds are taken into account individually in the estimation of esds in distances, angles and torsion angles; correlations between esds in cell parameters are only used when they are defined by crystal symmetry. An approximate (isotropic) treatment of cell esds is used for estimating esds involving l.s. planes.
Refinement. Refinement of F^2^ against ALL reflections. The weighted R-factor wR and goodness of fit S are based on F^2^, conventional R-factors R are based on F, with F set to zero for negative F^2^. The threshold expression of F^2^ > 2sigma(F^2^) is used only for calculating R-factors(gt) etc. and is not relevant to the choice of reflections for refinement. R-factors based on F^2^ are statistically about twice as large as those based on F, and R- factors based on ALL data will be even larger.The positions of all NH and OH hydrogen atoms were refined, and all CH were placed at ideal positions.

### Fractional atomic coordinates and isotropic or equivalent isotropic displacement parameters (Å^2^)

**Table:**

	*x*	*y*	*z*	*U*_iso_*/*U*_eq_	
C2	0.5660 (3)	0.1533 (2)	−0.12552 (17)	0.0484	
C4	0.5449 (3)	0.1836 (2)	0.0688 (2)	0.0626	
C5	0.6271 (4)	0.1200 (3)	0.1556 (2)	0.0725	
C6	0.7293 (3)	0.0372 (3)	0.1756 (2)	0.0728	
C7	0.7524 (3)	0.0132 (3)	0.1090 (2)	0.0682	
C8	0.6705 (3)	0.0761 (2)	0.02241 (18)	0.0503	
C9	0.5691 (2)	0.1616 (2)	0.00250 (16)	0.047	
C12	0.9604 (2)	0.2887 (2)	0.21087 (16)	0.047	
C14	0.8306 (3)	0.3839 (3)	−0.0275 (2)	0.0719	
C15	0.7199 (4)	0.4642 (3)	−0.0859 (2)	0.0819	
C16	0.6467 (4)	0.5164 (3)	−0.0557 (2)	0.0786	
C17	0.6837 (3)	0.4930 (3)	0.03457 (18)	0.0617	
C18	0.7951 (2)	0.4131 (2)	0.09334 (16)	0.0479	
C19	0.8659 (3)	0.3583 (2)	0.06268 (17)	0.0521	
H1	0.700 (3)	0.023 (3)	−0.073 (2)	0.0781*	
H3	0.440 (3)	0.256 (3)	−0.122 (2)	0.0603*	
H4	0.476	0.2396	0.0553	0.0818*	
H5	0.6134	0.1333	0.2013	0.0945*	
H6	0.7819	−0.0041	0.2343	0.0851*	
H7	0.82	−0.0425	0.1215	0.0843*	
H11	0.830 (3)	0.384 (3)	0.220 (2)	0.0595*	
H13	1.021 (3)	0.235 (3)	0.136 (2)	0.0682*	
H14	0.8797	0.3482	−0.0463	0.0925*	
H15	0.6943	0.4849	−0.1462	0.0986*	
H16	0.5679	0.568	−0.0992	0.0857*	
H17	0.6367	0.5309	0.0569	0.0697*	
H101	0.580 (3)	0.137 (3)	−0.227 (2)	0.0811*	
H102	0.472 (3)	0.237 (3)	−0.247 (2)	0.0806*	
H201	1.041 (3)	0.247 (3)	0.339 (2)	0.07*	
H202	1.115 (3)	0.183 (3)	0.301 (2)	0.0698*	
H261	0.979 (7)	0.918 (6)	0.069 (5)	0.1811*	
H262	0.925 (6)	0.838 (6)	0.005 (5)	0.1804*	
N1	0.6657 (2)	0.0745 (2)	−0.05947 (16)	0.0562	
N3	0.5078 (2)	0.20819 (19)	−0.08953 (14)	0.0477	
N10	0.5301 (3)	0.1737 (2)	−0.21296 (17)	0.0601	
N11	0.85749 (19)	0.3685 (2)	0.18649 (14)	0.0479	
N13	0.9688 (2)	0.2826 (2)	0.13810 (14)	0.0539	
N20	1.0417 (2)	0.2263 (2)	0.29315 (16)	0.0582	
O22	0.72767 (16)	0.64914 (16)	0.21275 (11)	0.0506	
O23	0.72874 (19)	0.44104 (15)	0.26788 (13)	0.0584	
O24	0.8960 (2)	0.5933 (2)	0.37860 (12)	0.0746	
O25	0.6554 (2)	0.61476 (17)	0.31313 (14)	0.0657	
O26	0.9603 (4)	0.8443 (3)	0.06107 (18)	0.1136	
S21	0.75054 (6)	0.57563 (5)	0.29273 (4)	0.0431	

### Atomic displacement parameters (Å^2^)

**Table:**

	*U*^11^	*U*^22^	*U*^33^	*U*^12^	*U*^13^	*U*^23^
C2	0.0604 (14)	0.0435 (12)	0.0537 (13)	0.0027 (10)	0.0411 (12)	−0.0021 (10)
C4	0.0850 (19)	0.0550 (15)	0.0668 (16)	0.0109 (13)	0.0563 (16)	0.0013 (12)
C5	0.109 (2)	0.0657 (17)	0.0641 (17)	0.0001 (17)	0.0640 (18)	0.0001 (14)
C6	0.087 (2)	0.0733 (18)	0.0555 (15)	0.0049 (16)	0.0420 (16)	0.0142 (14)
C7	0.0703 (17)	0.0651 (17)	0.0705 (17)	0.0194 (14)	0.0436 (15)	0.0185 (14)
C8	0.0577 (14)	0.0464 (12)	0.0551 (14)	0.0060 (11)	0.0388 (12)	0.0017 (11)
C9	0.0580 (13)	0.0411 (11)	0.0484 (12)	0.0015 (10)	0.0358 (11)	−0.0010 (10)
C12	0.0421 (11)	0.0541 (13)	0.0449 (12)	−0.0005 (10)	0.0266 (11)	−0.0062 (11)
C14	0.086 (2)	0.087 (2)	0.0555 (16)	0.0163 (16)	0.0498 (16)	−0.0027 (14)
C15	0.107 (3)	0.085 (2)	0.0498 (15)	0.0272 (19)	0.0461 (17)	0.0063 (14)
C16	0.090 (2)	0.0757 (19)	0.0485 (15)	0.0291 (17)	0.0315 (15)	−0.0001 (14)
C17	0.0615 (15)	0.0649 (16)	0.0475 (14)	0.0171 (13)	0.0277 (12)	−0.0065 (12)
C18	0.0467 (12)	0.0520 (13)	0.0425 (12)	0.0014 (10)	0.0261 (11)	−0.0085 (10)
C19	0.0542 (14)	0.0561 (14)	0.0480 (13)	0.0051 (11)	0.0322 (12)	−0.0054 (11)
N1	0.0716 (14)	0.0522 (12)	0.0658 (13)	0.0182 (11)	0.0527 (12)	0.0089 (10)
N3	0.0555 (11)	0.0470 (10)	0.0480 (11)	0.0102 (9)	0.0356 (10)	0.0018 (9)
N10	0.0807 (16)	0.0608 (13)	0.0583 (13)	0.0140 (11)	0.0525 (13)	0.0042 (10)
N11	0.0440 (10)	0.0592 (12)	0.0450 (10)	0.0050 (9)	0.0296 (9)	−0.0045 (9)
N13	0.0522 (12)	0.0660 (13)	0.0499 (11)	0.0139 (10)	0.0345 (10)	−0.0004 (10)
N20	0.0551 (12)	0.0685 (14)	0.0502 (12)	0.0098 (11)	0.0318 (11)	0.0020 (11)
O22	0.0481 (9)	0.0669 (10)	0.0421 (8)	0.0040 (7)	0.0302 (8)	0.0120 (7)
O23	0.0752 (12)	0.0507 (9)	0.0791 (12)	−0.0031 (8)	0.0624 (11)	−0.0046 (8)
O24	0.0619 (11)	0.1028 (15)	0.0410 (9)	−0.0251 (11)	0.0223 (9)	0.0111 (9)
O25	0.0948 (14)	0.0562 (10)	0.0907 (13)	0.0093 (9)	0.0796 (12)	0.0092 (9)
O26	0.144 (2)	0.148 (3)	0.0652 (14)	−0.069 (2)	0.0725 (17)	−0.0286 (16)
S21	0.0483 (3)	0.0508 (3)	0.0396 (3)	−0.0046 (3)	0.0317 (3)	0.0008 (2)

### Geometric parameters (Å, º)

**Table:**

C2—N1	1.336 (3)	C15—H15	0.925
C2—N3	1.333 (3)	C16—C17	1.374 (4)
C2—N10	1.326 (3)	C16—H16	0.95
C4—C5	1.380 (4)	C17—C18	1.379 (3)
C4—C9	1.377 (3)	C17—H17	0.955
C4—H4	0.931	C18—C19	1.387 (3)
C5—C6	1.379 (4)	C18—N11	1.393 (3)
C5—H5	0.92	C19—N13	1.389 (3)
C6—C7	1.378 (4)	H1—N1	0.80 (3)
C6—H6	0.922	H3—N3	0.83 (3)
C7—C8	1.373 (4)	H11—N11	0.85 (3)
C7—H7	0.921	H13—N13	0.83 (3)
C8—C9	1.390 (3)	H101—N10	0.87 (3)
C8—N1	1.396 (3)	H102—N10	0.89 (3)
C9—N3	1.386 (3)	H201—N20	0.83 (3)
C12—N11	1.343 (3)	H202—N20	0.93 (3)
C12—N13	1.338 (3)	H261—O26	0.81 (6)
C12—N20	1.321 (3)	H262—O26	0.79 (6)
C14—C15	1.376 (4)	O22—S21	1.4723 (15)
C14—C19	1.385 (4)	O23—S21	1.4711 (18)
C14—H14	0.919	O24—S21	1.4680 (19)
C15—C16	1.394 (4)	O25—S21	1.4596 (16)
			
N1—C2—N3	109.0 (2)	C16—C17—H17	122.5
N1—C2—N10	125.7 (2)	C18—C17—H17	120.8
N3—C2—N10	125.2 (2)	C17—C18—C19	121.7 (2)
C5—C4—C9	117.5 (2)	C17—C18—N11	131.6 (2)
C5—C4—H4	121.5	C19—C18—N11	106.7 (2)
C9—C4—H4	121	C18—C19—C14	121.6 (2)
C4—C5—C6	121.5 (3)	C18—C19—N13	106.5 (2)
C4—C5—H5	119.2	C14—C19—N13	131.8 (2)
C6—C5—H5	119.3	C8—N1—C2	109.08 (19)
C5—C6—C7	121.3 (3)	C8—N1—H1	127 (2)
C5—C6—H6	119.3	C2—N1—H1	122 (2)
C7—C6—H6	119.4	C9—N3—C2	109.2 (2)
C6—C7—C8	117.2 (3)	C9—N3—H3	128.6 (19)
C6—C7—H7	122	C2—N3—H3	121.9 (19)
C8—C7—H7	120.8	H102—N10—C2	117 (2)
C7—C8—C9	121.8 (2)	H102—N10—H101	124 (3)
C7—C8—N1	132.3 (2)	C2—N10—H101	117 (2)
C9—C8—N1	106.0 (2)	C18—N11—C12	108.65 (18)
C8—C9—C4	120.7 (2)	C18—N11—H11	125.7 (19)
C8—C9—N3	106.76 (19)	C12—N11—H11	125.5 (19)
C4—C9—N3	132.5 (2)	C19—N13—C12	109.13 (19)
N11—C12—N13	109.0 (2)	C19—N13—H13	125 (2)
N11—C12—N20	126.2 (2)	C12—N13—H13	126 (2)
N13—C12—N20	124.8 (2)	H202—N20—C12	114.7 (18)
C15—C14—C19	116.6 (2)	H202—N20—H201	124 (3)
C15—C14—H14	122.8	C12—N20—H201	118 (2)
C19—C14—H14	120.7	H261—O26—H262	100 (6)
C14—C15—C16	121.6 (3)	O22—S21—O23	109.89 (10)
C14—C15—H15	119.2	O22—S21—O24	108.29 (10)
C16—C15—H15	119.3	O23—S21—O24	108.23 (12)
C15—C16—C17	121.7 (3)	O22—S21—O25	111.26 (10)
C15—C16—H16	119.2	O23—S21—O25	108.86 (10)
C17—C16—H16	119.1	O24—S21—O25	110.26 (13)
C16—C17—C18	116.8 (2)		

### Hydrogen-bond geometry (Å, º)

**Table:**

*D*—H···*A*	*D*—H	H···*A*	*D*···*A*	*D*—H···*A*
N1—H1···O25^i^	0.81 (4)	2.25 (3)	2.946 (3)	144 (3)
N3—H3···O22^ii^	0.83 (3)	1.93 (3)	2.749 (3)	172 (3)
N11—H11···O23	0.85 (4)	1.96 (4)	2.786 (4)	166 (3)
N13—H13···O24^iii^	0.83 (4)	1.91 (4)	2.720 (3)	165 (3)
N10—H101···O23^i^	0.87 (4)	2.03 (4)	2.894 (5)	169 (3)
N10—H102···O25^ii^	0.89 (3)	2.00 (3)	2.890 (3)	175 (3)
N20—H201···O26^iii^	0.84 (3)	2.04 (3)	2.853 (4)	165 (3)
N20—H202···O22^iii^	0.93 (4)	2.09 (4)	2.973 (4)	157 (3)
O26—H261···O24^iv^	0.80 (7)	2.22 (7)	2.983 (4)	160 (7)
O26—H262···O24^v^	0.80 (7)	2.14 (7)	2.860 (4)	150 (6)
C17—H17···O22	0.95	2.56	3.272 (3)	132

Symmetry codes: (i) *x*, −*y*+1/2, *z*−1/2; (ii) −*x*+1, −*y*+1, −*z*; (iii) −*x*+2, *y*−1/2, −*z*+1/2; (iv) −*x*+2, *y*+1/2, −*z*+1/2; (v) *x*, −*y*+3/2, *z*−1/2.
